# Resolving Acuticulata (Metridioidea: Enthemonae: Actiniaria), a clade containing many invasive species of sea anemones

**DOI:** 10.1371/journal.pone.0328544

**Published:** 2025-08-14

**Authors:** Isabel A. M. Pen, Charlotte Benedict, Michael B. Broe, Alonso Delgado, Heather Glon, Mengjin Zhang, Marymegan Daly

**Affiliations:** 1 Department of Evolution, Ecology, and Organismal Biology, Ohio State University, Columbus, Ohio, United States of America; 2 Maine Department of Marine Resources, Bureau of Marine Science, West Boothbay Harbor, Maine, United States of America; 3 School of Biological Sciences, University of Hong Kong, Hong Kong SAR, P. R. China; Laboratoire de Biologie du Développement de Villefranche-sur-Mer, FRANCE

## Abstract

Acuticulata is a globally distributed group in the actiniarian superfamily Metridioidea comprised of taxa with ecological, economic, and scientific significance. Prominent members such as *Exaiptasia diaphana* and *Diadumene lineata* serve as model organisms for studying coral symbiosis, bleaching phenomena, and ecological invasions. Despite their importance, unresolved phylogenetic relationships and outdated taxonomic frameworks hinder a full understanding of the diversity and evolution of the taxa in this clade. In this study, we employ a targeted sequence-capture approach to construct a robust phylogeny for Acuticulata, addressing long-standing questions about familial monophyly and comparing the results to results from a more conventional five-gene dataset. Specimens from previously underrepresented families and global regions, including the Falkland Islands, were included to elucidate evolutionary interrelationships and improve resolution. Our results support the monophyly of Aliciidae, Boloceroididae, Diadumenidae, Gonactiniidae, and Metridiidae. Our results reiterate the need for taxonomic revision within the family Sagartiidae, as the specimens we included from this family were recovered in four distinct clades. Based on our results, we transfer *Paraiptasia* from Aiptasiidae to Sagartiidae. These findings emphasize the utility of genome-scale data for resolving phylogenetic ambiguities for morphologically problematic taxa and suggest a framework for future integrative taxonomic and ecological studies within Acuticulata.

## Introduction

With members found in all the world’s oceans, the acuticulate clade in the actiniarian superfamily Metridioidea Carlgren, 1893 holds significant economic, scientific, and ecological importance. Its most well-known member is the notorious aquarium pest, the pale anemone, *Exaiptasia diaphana* (Rapp, 1829) [1] (still commonly referred to as its junior synonyms *Aiptasia pallida* and *Exaiptasia pallida*), that often overtakes substrates and outcompetes ornamental aquarium organisms. This species has also been pivotal in the investigation of coral symbiosis as a model organism for testing hypotheses about bleaching phenomena [2–6]. Additionally, the genome of *E. diaphana* was sequenced and annotated a decade ago [7], which has led to its use in the fields of molecular biology [8], regeneration biology [9], and environmental stress physiology [10–14]. Another prominent member of the acuticulate clade, the orange-striped green sea anemone, *Diadumene lineata* (Verrill, 1869) [15], is rapidly emerging as a valuable model for studying a variety of biological and ecological phenomena, including nematocyst discharge and ion channel activity [16–18], as well as plasticity in clonal growth and reproduction in response to environmental conditions [19–22]. Research on *D. lineata* also highlights its role in ecological invasions, including substrate preferences [23], potential invasional meltdowns [24], salinity tolerance and distributional limits [25], as well as predictive modeling of its distribution and establishment in non-native environments [26].

The pervasive issue of marine biological invasions, exacerbated by increasing anthropogenic dispersal and environmental changes, presents a growing challenge for marine ecosystems globally. Actiniarians in superfamily Acuticulata play critical roles in marine ecosystems, maintaining the ecological balance in their native ranges and often disrupting ecosystems in their introduced ranges by altering habitats through feeding and settlement behaviors. Among the members of the acuticulate clade of Metridioidea, *Exaiptasia diaphana*, *Diadumene lineata*, and *Metridium senile* (Linnaeus, 1761) [27] thrive in human-created habitats, such as vessels, marinas, and aquaculture sites, due to their euryhaline and eurythermal tendencies [28]. Many invasive sea anemone species (reviewed in [28]) exhibit both sexual and asexual reproduction, the latter by means of fission or pedal laceration, which enables rapid population establishment, particularly in disturbed or artificial environments. In their introduced ranges, these species can outcompete native organisms for resources, leading to shifts in species composition and disrupted trophic interactions. For example, the plumose sea anemone, *Metridium senile*, is known to form dense aggregations that dominate benthic communities and negatively impact biodiversity in these habitats. Invasive anemones also contribute to biofouling, attaching to ship hulls, docks, and aquaculture infrastructure [29,30]. Anthropogenic activities like global shipping have facilitated the dispersal of invasive sea anemone species in the acuticulate clade, convoluting conclusions on their historical biogeography. Various reproductive strategies and dispersal methods, including asexual reproduction and long-lived planktonic larvae, have likely contributed to their widespread distribution and ability to tolerate a broad range of ecological variables. Without a well-resolved phylogeny of these taxa within the superfamily, it is difficult to draw conclusions about the evolution of these traits or take full advantage of the potential embodied by the genomic, physiological, and ecological resources that have been developed for invasive *Exaiptasia* Grajales & Rodríguez, 2014 [31] or *Diadumene* Stephenson, 1920 [32]. It is unclear if attributes linked with invasiveness represent multiple independent evolutions or are the ancestral state for the superfamily.

Historically, members of the superfamily Metridioidea have been unified by a general lack of morphological characters by which to piece together their evolutionary relationships [33]. The few diagnostic characters for this group, all of which are internal, are the presence of mesenteries, or vertical sheets of endoderm-derived tissue that extend into the body wall, that are divisible into larger, primary mesenteries that house reproductive structures (macrocnemes) and smaller, secondary mesenteries (microcnemes). Most acuticulate metridioideans also possess a relatively weak mesogleal marginal sphincter muscle and have defensive structures called acontia, nematocyst-dense, thread-like extensions of the mesenterial filaments that may be extruded from the column when the anemone is stressed or threatened [34]. These three characters may be more appropriately interpreted as shared features of Metridioidea, and the latter two characters are inferred to have been lost in multiple lineages [33], which make it challenging to identify clade membership. Nematocyst composition has the potential to serve as a diagnostic tool for identifying taxa within this clade, however the interpretation of nematocyst morphology, particularly of those found in the acontia, present challenges due to their small size and complexity [35–40]. The lack of distinct morphological features, combined with superficial similarity due to secondary losses (i.e., *Metridium* de Blainville, 1824 [41] and *Diadumene*) has convoluted the understanding of relationships among members of the acuticulate clade of Metridioidea based on morphology alone.

Within the acuticulate clade, the family Sagartiidae Gosse, 1858 [42] has historically been the family to which most species with acontia have been assigned. This family has persistent taxonomic issues, particularly surrounding the genus *Sagartia* Gosse, 1855 [43]. Historically, *Sagartia* has served as a “recycling bin” genus into which many species that generally align with the morphological diagnosis of the acuticulate clade were placed. The lack of clear, unifying diagnostic characters resulted in an assortment of taxa being assigned to *Sagartia*, despite considerable variation in their morphological and ecological traits. The genus lacked a stable taxonomic framework and many species placed in it were either poorly defined or were later recognized as belonging to different lineages. Sanamyan and Sanamyan [44] revisited an earlier evaluation of the genus [45], which listed all sagartiid genera as *incertae sedis* and treated this family as invalid, while simultaneously proposing no solution. A historical misinterpretation of type species designations, as a result of the large number of sea anemone species that were originally described in the genus *Actinia* Linnaeus, 1767 [46] further complicated the situation. Following the International Code of Zoological Nomenclature, Sanamyan and Sanamyan [44] moved species previously assigned to the genus *Sagartia* to *Cylista* Wright, 1859 [47] and designated *Actinia* (now *Cylista*) *trogolodytes* (Price in Johnston, 1847) [48] as the type species of that genus. They also noted the presence of two distinct groups of species within the sagartiid genus *Sagartiogeton* Carlgren, 1924 [49] and therefore moved three species previously assigned to *Sagartiogeton* into *Sagartia,* recognizing *Sagartia*, *Sagartiogeton,* and *Cylista* as valid genera [44]. These taxonomic revisions stabilized the genera involved, the monophyly and taxonomic status of the family Sagartiidae remains unclear.

The monogeneric family Diadumenidae Stephenson, 1920 [32], also within the acuticulate clade, is primarily composed of small-bodied sea anemone species that have demonstrated remarkable affinity for human-modified environments. At least four species documented to be globally-invasive in the genus *Diadumene* are easily transported across oceans in ballast water and hull fouls, exhibiting resilience to a variety of environmental conditions and spreading quickly through rapid asexual reproduction via pedal laceration [28]. The taxonomic history of this family is complex because many species were spuriously described as new species from invasive populations outside their native ranges [50]. This leaves room for uncertainty about the geographic origins and validity of several species. The fact that type localities may not represent the true native ranges, along with over a century of historical misidentifications and synonymizations, complicates their taxonomy and poses challenges for understanding the biogeography and evolutionary history for members of this family.

Cryptic diversity also complicates taxonomy within Actiniaria and is particularly prevalent in the acuticulate clade [51–53]. Because invasive taxa are widely distributed, some species have been mistakenly described as multiple distinct species, complicating their classification and hindering taxonomic revision efforts. Cryptic diversity also exists within taxa not recognized as invasive, such as *Boloceroides mcmurrichi* (Kwietniewski, 1898) [54]. This species is currently recognized as circum-globally distributed, however recent studies have demonstrated that this likely represents a species complex [55].

The most recent comprehensive study of actiniarian systematics analyzed a five-gene dataset (two nuclear and three mitochondrial genes), to subdivide Actiniaria into three suborders and five superfamilies, including the superfamily Metridioidea [35]. While five-gene datasets are widely available, affordable, and relatively easy to sequence, these markers are insufficiently variable to resolve closely related species and show limited support for deep branches within each superfamily. In clades like Metridioidea, family-level groupings, which are well established and supported in other taxa (e.g., Mammalia, Insecta), remain unresolved within much of this clade under the five-gene approach [35]. However, high-fidelity, genome-level sequencing, has led to the development of sequence-capture baits targeting conserved loci [56,57] that provide a data-rich alternative to the five-gene approach. Although more resource-intensive in terms of cost and computational demands, this method is particularly powerful for disentangling taxa where morphology does not point to one clear conclusion [58,59], and, for anemones, has highlighted diversity that was not apparent under a conventional, five-gene approach. The broader and deeper genetic coverage offered by targeted sequence capture loci makes it a superior tool for resolving fine-scale phylogenetic relationships that remain elusive with traditional five-gene datasets.

In this study, data from both the standard five-gene dataset and targeted sequence capture are employed to generate robust phylogenies for the acuticulate clade of the superfamily Metridioidea. These two datasets are used in tandem to resolve remaining questions about familial monophyly and interrelationships among taxa of economic and scientific importance. We also use this opportunity to follow the methods of Benedict et al. [55] to identify specimens of acuticulate actiniarians collected in the Falkland Islands (Malvinas).

## Materials and methods

### Specimen collection and DNA extraction

Actiniarian specimens were collected either by hand, while SCUBA diving, or via trawls. Specimens were preserved in 95% ethanol to ensure integrity of molecular data. Specimens were identified using polyp anatomy and cnidae and labeled as “indet” when precise species identification was not possible. Twelve specimens from the Falkland Islands were collected by hand while SCUBA diving (maximum depth of 20 m) during the 2019 research cruise for the “Fine Scaling of the Marine Management Area of the Falkland Islands” project. Collection data for each specimen is contained in S1 Appendix, with permit information provided for samples collected by the authors.

DNA extraction was performed using the E.Z.N.A. Tissue DNA Kit (Omega Bio-Tek, Norcross, GA, USA) according to the manufacturer’s protocol, except with a reduced elution volume to increase concentration. The quality and quantity of extracted DNA were evaluated using a Qubit 2.0 fluorometer and a NanoDrop spectrophotometer, measuring DNA concentration (ng/μL). Specimens with remaining tissue have been deposited at the American Museum of Natural History (AMNH). Taxa sampled represent 75% of the acuticulate diversity at the family-level (9 of 12 valid families), and include several unidentified individuals collected from the Falkland Islands in 2019. No members of Haliactinidae Carlgren, 1949 [60], Haliplanellidae Hand, 1956 [61], nor Sagartiomorphidae Carlgren, 1934 [62] were considered in the phylogenetic framework presented here.

### Library preparation

Up to 1000 ng of DNA per sample was used for library preparations, which were conducted using the Kapa HyperPrep Kit (Kapa Biosciences, Wilmington, MA, USA), optimized for target capture. Universal Y-yoke oligonucleotide adapters and iTru dual-indexed primers were used as described in Glenn et al. [63]. Eleven libraries were pooled in equimolar ratios, each contributing approximately 100 ng, for a total of 1.3–2.3 μg of DNA per pool for target enrichment.

### Target enrichment and sequencing

Target enrichment and sequencing were conducted at Arbor Biosciences (Ann Arbor, MI, USA). The hexacoral-v2 bait set [56] used contained a total of 25,288 baits targeting 2,476 (1,127 UCE and 1,349 exon) loci. Target enrichment was conducted according to the MyBaits v.IV protocol with bait concentrations of 500 ng per reaction. Libraries were sequenced using 150 bp paired-end sequencing on an Illumina NovaSeq platform. Resulting newly generated sequences are available on NCBI GenBank (PRJNA1250587).

### Post-sequencing dataset assembly

Paired-end reads were trimmed to remove low-quality bases and adapters using TrimGalore [64]. Trimmed reads were then assembled into contigs using SPAdes genome assembler v3.15.5 [65]. To maximize taxon representation, transcriptome data procured from NCBI GenBank were included, prioritizing it over the total number of loci recovered. These transcriptomic data were then aligned with contigs generated for this study and processed simultaneously. The PHYLUCE UCE Data Preparation Pipeline [66] was used to extract loci, and loci were matched to the bait set using the phyluce_assembly_match_contigs_to_probes command [66], with a minimum coverage threshold of 70% and a minimum identity threshold of 70% for increased accuracy. Additionally, Gblocks [67] was used for internal trimming under default parameters to refine alignments. Target capture loci were then extracted and taxon occupancy matrices were generated for both 50% and 75% completeness using the phyluce_align_get_only_loci_with_min_taxa command [66]. These were concatenated and aligned with our contigs and processed simultaneously. Sequences from 73 individuals were used to generate final matrices, with *Stomphia didemon* Siebert, 1973 [68] used as an outgroup to root the tree. Annotated code used in this analysis is available from the Dryad Digital Repository (10.5061/dryad.vmcvdnd32).

### Phylogenetic analyses of target capture loci

Maximum likelihood trees were constructed using IQ-TREE v2.2.0 [69] employing the best-fit model with lowest BIC selected automatically by ModelFinder with 1000 ultrafast bootstraps. To further refine phylogenetic relationships, additional maximum likelihood analyses were conducted using RAxML [70]. Multiple sequence alignments were prepared, checking for gaps and invariant sites. Maximum likelihood trees were inferred using RAxML with the GTR + G model, incorporating empirical base frequencies (GTR + FU + G) and accounting for rate variation across sites with a gamma distribution. For robustness, 25 parsimony and 25 random starting trees were used. Bootstrap analyses were performed to assess the reliability of the inferred phylogenies, using 1,000 bootstrap replicates for each dataset. Convergence of bootstrap replicates was tested, with a cutoff of 0.03 (3%). Final branch support values were mapped onto the best-scoring trees generated. UCE and exon loci were not separated following previous studies [56,57,71,72].

Multiple sequence alignments for neighbor-joining trees were processed using TrimAL v1.5.rev0 [73] to remove poorly aligned positions and reduce alignment noise. A gap threshold of 0.8 was applied, retaining only columns where at least 80% of sequences had data. The resulting filtered alignment was used for phylogenetic inference. Neighbor-joining trees were constructed using IQ-TREE v2.2.0.3 [69] with the balanced neighbor-joining (BIONJ) algorithm. The GTR + FU + G substitution model was applied to compare to the maximum likelihood trees.

### Phylogenetic analyses of five-gene loci

In addition to the phylogenetic analysis of target capture loci, a tree was generated from two nuclear (18S, 28S) and three mitochondrial (12S, 16S, *cox*3) genes, following Rodríguez et al. [35], to compare the grouping information and assess the effect of taxon sampling on tree topology (S1 Fig). A total of 99 samples were included in the analysis. Sequences for 84 samples were retrieved from GenBank based on Rodríguez et al. [35] and Grajales & Rodríguez [53]. An additional 15 samples were newly sequenced and incorporated into this analysis. To ensure consistency in sequence quality and length, newly extracted sequences with excessive ambiguity were trimmed and some previously generated sequences were shortened manually in Geneious Prime 2022.1.1 [74] to achieve comparable lengths across the dataset. The concatenated sequences were aligned using MUSCLE [75] and phylogenetic analyses were performed using Maximum Likelihood (ML) in IQ-TREE [76]. The TN93 + F + G4 model was used, as determined by model selection. The data were subjected to 1000 ultrafast bootstrap alignments, with 1000 iterations and 200 as the stopping rule.

## Results

### Read, assembly, and locus recovery statistics

The total number of raw reads ranged from 596,140–12,527,559 per sample (mean: 4,361,194 ± 2,136,820 SD) within the 59 target capture samples. After removing adapter contamination and low-quality bases by trimming with TrimGalore [64], the number of reads per sample decreased by 0.68%, resulting in a mean of 4,331,369.69 ± 2,125,185.56 SD trimmed reads per sample. The reduction in reads ranged from 0.82% to 1.29% per sample. These trimmed reads were then assembled into contigs using SPAdes genome assembler v3.15.5 [65], generating a range of 9,600–248,454 contigs per sample (mean: 68,623.91 ± 71,233.30 bp). In total, 714 loci (3,033 targeted) were recovered from the assembled contigs. Mean length of target capture loci was 632.71 ± 270.89 bp, ranging from 228 to 1828 bp.

### Alignment statistics

Two alignment matrices were generated using the PHYLUCE UCE Data Preparation Pipeline [66], with sequences aligned using MAFFT [77]. We considered two matrices that differed in the average number of taxa with sequence data for each locus: a 50% occupancy matrix that contained the sequence data for all loci present in at least 50% of the samples, and a 75% occupancy matrix, which contained the sequence data for all loci present in at least 75% of the samples. The 50% occupancy matrix included 714 loci with a trimmed mean locus length of 633 bp, totaling 451,752 bp. The 75% occupancy matrix included nine loci with a trimmed mean locus length of 828 bp, totaling 7,456 bp. The 50% matrix contained 289,176 informative sites, averaging 405 per locus. The average number of taxa per locus was 41 at 50% occupancy and 56 at 75% occupancy, with a range of 36–59 taxa across both matrices.

### Phylogenetic analysis of five-gene loci

The tree from analysis of the five-gene dataset finds a monophyletic Aliciidae Duerden, 1895 [78] sister to a clade containing Boloceroididae Carlgren, 1924 [79] and Gonactiniidae Carlgren, 1893 [80]. All members of Aiptasiidae Carlgren, 1924 [81] are part of the same clade, sister to the Aliciidae-Boloceroididae-Gonactiniidae clade. Some specimens initially identified as *Diadumene* were grouped with *Aiptasiogeton* Schmidt, 1972 [37], indicating possible misidentifications; these same samples were recovered in the Aiptasiidae clade in the target capture analyses. *Aiptasiogeton*, a genus whose members are non-symbiotic, was recovered as sister taxa to the model organism *Exaiptasia diaphana*. Sagartiidae is not recovered as a monophyletic group: members of this family are distributed throughout the tree (see S1 Fig), similar to findings in Rodríguez et al. [35].

### Phylogenetic analysis of target capture loci

**Fig 1:**
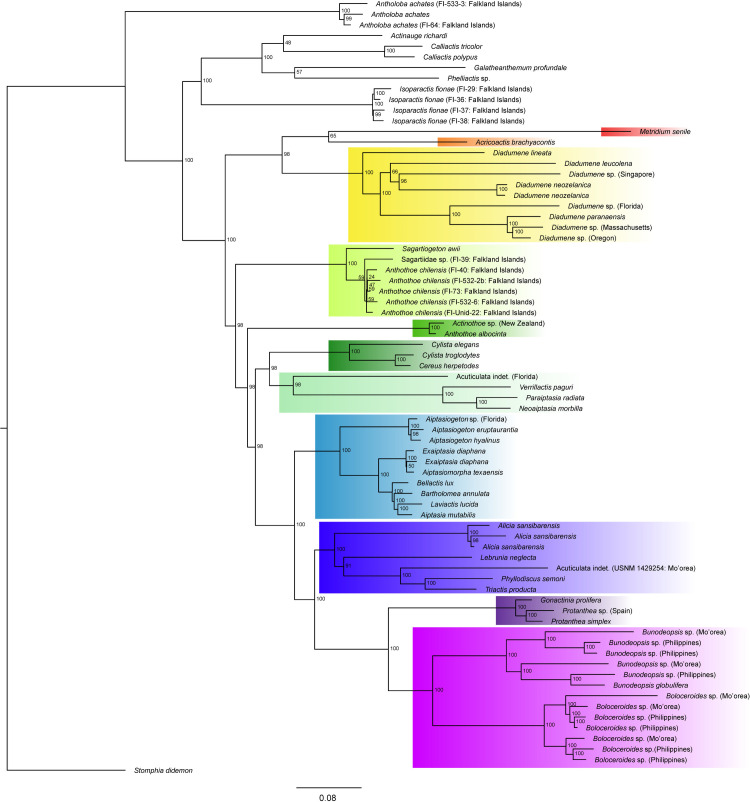
Phylogenetic reconstruction of Acuticulata using target capture loci. Maximum likelihood phylogenetic tree based on the 50% data matrix (714 loci, 451,752 bp). Ultrafast bootstrap values as shown at nodes. Taxa color-coded by family-level grouping: Metridiidae – red, Acricoactinidae – orange, Diadumenidae – yellow, Sagartiidae I – lime green, Sagartiidae II – kelly green, Sagartiidae III – forest green, Sagartiidae IV – mint green, Aiptasiidae – cerulean, Aliciidae – indigo, Gonactiniidae – violet, Boloceroididae – fuchsia.

Phylogenetic analyses were performed using IQ-TREE2 [69] and RAxML [70] on the 50% and 75% occupancy datasets. The maximum likelihood topologies were largely congruent between both the datasets and methods. Bootstrap support values were slightly higher for the 50% dataset, with the majority of values reaching 100% (Fig 1). Results of neighbor-joining analysis recovered an identical topology to those of the maximum likelihood analysis for ingroup taxa, except for minor differences in the placement of some specimens of *Anthothoe chilensis* (Lesson, 1830) [82] within the species clade (S2 Fig; lime green).

Members of the cuticulate clade did not resolve as monophyletic, with the family Antholobidae Durán-Fuentes, González-Muñoz, Daly & Stampar, 2024 [83] recovered as sister to a clade containing the other members of the cuticulate clade and all members of the acuticulate clade sampled. However, results support the monophyly of the acuticulate clade. Two major clades were recovered: a clade containing members of the families Acricoactinidae Larson, 2016 [84], Metridiidae Carlgren, 1893 [80], and Diadumenidae (hereafter referred to as Metridina), sister to a clade containing members of Sagartiidae, Aiptasiidae, Aliciidae, Boloceroididae, and Gonactiniidae (hereafter referred to as Sagartina). All of the aforementioned families were recovered as monophyletic with the exception of Sagartiidae, which was composed of four groups, differentiated here with Roman numerals. Within Sagartina, Boloceroididae was recovered as sister to Gonactiniidae, with that group recovered as sister to Aliciidae. That clade of three families (hereafter referred to as the ABG clade) was recovered as sister to Aiptasiidae. Aiptasiidae + the ABG clade were recovered as sister to a clade comprised of Sagartiidae (III) and Sagartiidae (IV). All of the aforementioned families were sister to Sagartiidae (II), and that larger group is sister to Sagartiidae (I). Sagartiidae (I) includes *Anthothoe chilensis* along with several unidentified specimens from the Falkland Islands. The unidentified individuals from the Falkland Islands resolved within three clades: a clade containing *Anthothoe chilensis* and *Sagartiogeton awii* (Rodríguez, López-González & Daly, 2009) [85], a clade containing *Isoparactis fionae* Lauretta, Häussermann, Brugler & Rodríguez, 2014 [86], and one with *Antholoba achetes* (Drayton in Dana, 1846) [87].

## Discussion

### Ecological implications

The acuticulate clade of Metridioidea is comprised of taxa primarily found to inhabit intertidal and shallow subtidal habitats and attach to substrates (i.e., non-burrowing). Life in this fluctuating environment may predispose species in the acuticulate clade to success as invasive species [28]. Both Metridina (see Rodríguez et al. [33]) and Sagartina contain both known invasive taxa and taxa not known to be invasive [28]. These ecotypes appear to cluster in the tree, with Acricoactinidae, the ABG clade and Sagartiidae I, II, and IV having no members known to be invasive and Aiptasiidae, Diadumenidae, Metridiidae, and Sagartiidae III each containing at least one known invasive species. At a broad level, this result indicates that traits linked to invasiveness, such as asexual reproduction and broad ecological tolerance, have evolved independently (or undergone parallel losses) in multiple lineages within the acuticulate clade. A more direct investigation of the traits which allow these lineages to establish in new environments and spread within a more densely sampled phylogeny is necessary to more thoroughly test hypotheses on convergent evolution or exaptation.

One challenge in generating such a matrix of all species in this phylogeny is the incompleteness of original species descriptions and circumscriptions. Older descriptions, while often rich in natural history, lack detail on histology, nematocyst composition, or type locality. The modern standard for species descriptions includes detailed measurements of nematocyst composition, but ecological information beyond depth and substrate is uncommon. Understudied species are especially likely to have not been examined under the modern standard and to be subject to outdated taxonomy based on very few characters, whereas invasive or otherwise common species are more likely to have been revised or re-examined, even if only because they typically contain multiple junior synonyms. Invasive species tend to be easier to sample, and so are generally included in molecular phylogenies, whereas rare species, or species inhabiting difficult-to-sample habitats are less likely to be included. Widespread species, invasive or not, may also harbor cryptic diversity that complicates assessment of ecological traits and genetic divergence. For this reason, learning through earlier work that the diversity of Boloceroididae is not reflected in its taxonomy [55], we include many samples in the family Boloceroididae, which contains the circum-globally distributed genus *Bunodeopsis* and species complex *Boloceroides mcmurrichi*. This family was found to contain two well-supported clades for each genus. Within Aiptasiidae, the circum-Tropical West Atlantic *Bartholomea annulata* (Le Sueur, 1817) [88] includes at least two well-diverged, sister lineages [51] and the well-characterized invasive *Exaiptasia diaphana* was split to recognize the more geographically restricted and genetically distinct *Exaiptasia brasilensis* Grajales and Rodríguez 2016 [53], which also differs in its patterns of host association and habitat preference [52].

### Morphological limitations

The small size and lack of distinct external morphological features in many taxa within the acuticulate clade continues to impede accurate species identification. This limitation is particularly pronounced for species complexes, where genetically distinct lineages have not yet been studied closely enough to identify morphology by which to distinguish them (e.g., *Exaiptasia diaphana* and *E. brasilensis*, *Metridium senile* and *M. fimbriatum*: see [31,89]). As a result, there is still more work to do to fully assess the species level diversity for Acuticulata.

The dearth of external features complicates identification of taxa below the genus level; even experts may not feel comfortable identifying samples to species without examining nematocyst composition and internal morphology. The widespread use of platforms like iNaturalist introduces challenges, as non-expert users may overestimate their ability to correctly identify species, often resulting in spurious reports. This is particularly problematic when users incorrectly label new records with the names of known species, especially invasives. The reliance on external morphology in a photograph in non-standardized lighting and without scale can lead to misidentification, compromising data quality and hindering effective biodiversity monitoring. This highlights the need for an accurate sampling and description effort that integrates standardized molecular and morphological methods to circumscribe and validate the species in this clade (166 valid species in 46 valid genera: Rodríguez et al. [35]).

Because of their generally small stature and relatively streamlined anatomy, non-anatomical features like nematocysts are potentially promising for identifying acuticulate metridioideans [31,36,60,90]. However, at least some of the challenges for interpreting morphological diversity extend to the interpretation of nematocyst morphologies, particularly for those nematocysts found in acontia. In general, the relationships among morphologies of nematocysts are complex and poorly understood [35–40], with some aspects only visible in undischarged capsules under standard 100X compound microscopy that are difficult to differentiate. Current understanding of the distribution of cnidae suggests that *p*-mastigophores B2a are widespread among members of Metridioidea and rare or absent among actinioideans and anenthemonaens (see [35,91,92]). All species in Acuticulata for which cnidae have been documented through drawings or images (e.g., [36,61]) have capsules that would be identified as microbasic *p*-mastigophores/*p*-mastigophores B2a. In the case of Aiptasiidae, nematocyst size, particularly microbasic b-mastigophores/*p*-mastigophores B2a in the column, correlates with lineages defined by genetic data, and thus may serve as a potential diagnostic character for genus and family level grouping [53]. However, this correlation does not appear to be universal across all nematocyst types, and size can be a complicated metric [93,94] and thus should be considered just one tool in the taxonomic toolbox.

### Phylogenetic insights

**Fig 2:**
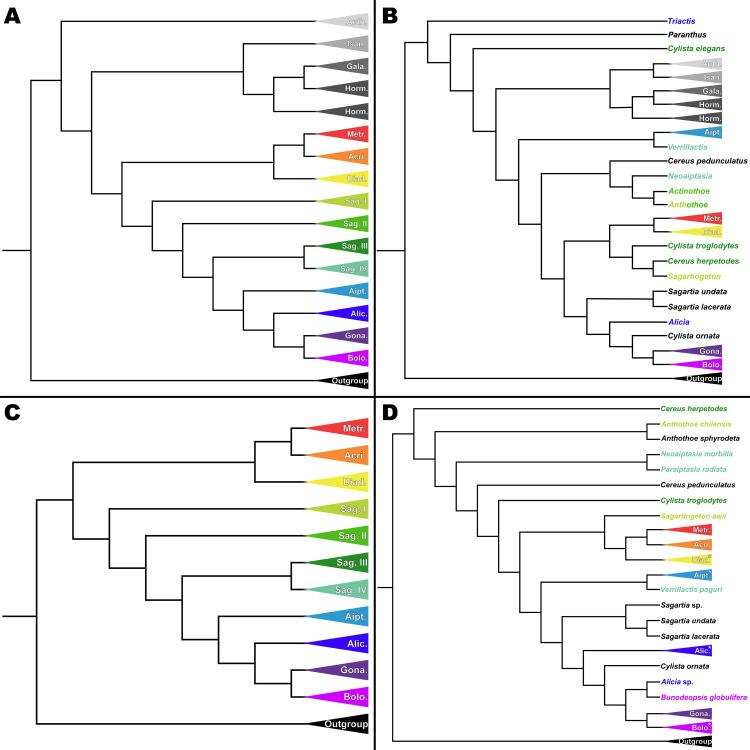
Phylogenetic comparisons of Acuticulata using genome-scale and five-gene datasets. Abbreviations for families are as follows (color): Anth. – Antholobidae (very light gray), Isan. – Isanthidae (light gray), Gala. – Galatheanthemidae (medium gray), Horm. – Hormathiidae (dark gray), Metr. – Metridiidae (red), Acri. – Acricoactinidae (orange), Diad. – Diadumenidae (yellow), Sag. – Sagartiidae (I – lime gren, II – kelly green, III – forest green, IV – mint green), Aipt. – Aiptasiidae (cerulean), Alic. – Aliciidae (indigo), Gona. – Gonactiniidae (violet), Bolo. – Boloceroididae (fuchsia). Taxa in (B) and (D) panels are colored based on the families in which they resolve in the genome-scale analysis (A and C). Taxa displayed in black text represent those not included in the target sequence dataset. (A) Simplified phylogeny of Metridioidea based on the target capture dataset, highlighting relationships within Acuticulata and some members of Cuticulata. (B) Simplified phylogeny based on Rodríguez et al. [35] five-gene dataset. (C) Simplified phylogeny restricted to acuticulate taxa based on the target capture dataset. (D) Simplified phylogeny generated in this study based on a five-gene dataset, with asterisks on Diadumenidae, Aiptasiidae, Aliciidae, and Boloceroididae indicating incomplete monophyly due to 1–3 outlier taxa (see S1 Fig).

The use of targeted sequence capture loci in this study significantly improved the resolution of phylogenetic relationships within Acuticulata. Compared to previous studies which considered all Actiniaria [35] and to a focused study of Acuticulata using a five-gene approach (S1 Fig), the target capture approach has increased bootstrap support (BS) values (Fig 1) and stronger resolution for difficult-to-place taxa. This is true even in cases where the five-gene data set has denser taxon sampling (either of the larger clade or of the ingroup) (Fig 2C and 2D), highlighting the benefit of genome-scale sampling over taxon sampling (reviewed in [95]). For actiniarians, our results demonstrate the utility of the approach of targeted sequence-capture loci, not only for fine-scale phylogenetic studies [71] or ordinal scale studies [96], but also for inquiries into the relationships of metridioidean taxa that are otherwise hard to resolve.

Genome scale approaches to reconstructing phylogeny offer the possibility of reconstruction and analysis of whole mitochondrial genomes [97–99]. We view this as an exciting additional benefit of target capture and genome skimming approaches but have not incorporated analysis of whole mitochondrial genomes into this dataset. Reconstructing mitogenomes for all of these taxa is a substantial undertaking, because of the number of taxa for which we provided new data and because the mitogenome of acuticulate actiniarians is not as straightforward to assemble as that of other lineages [100]. Given these challenges and the insight from previous studies showing limited power of those data for questions of interfamilial diversity [98,101,102], we have chosen to focus on the target capture and conventionally used markers at this time.

Though Bayesian inference is a powerful phylogenetic method that integrates across the posterior probability distribution to estimate tree topology, branch lengths, and model parameters, its application to this large-scale dataset is computationally prohibitive. Markov Chain Monte Carlo (MCMC) sampling is required for Bayesian tree search, but scales poorly with increasing sequence length and taxon number [103]. Bayesian analyses on datasets exceeding 100,000 sites can require months to years of computational time, even when utilizing high-performance computing clusters [104]. Given that the dataset in this study contains over 700 loci, the required MCMC chain length and sampling frequency would necessitate an impractical number of generations for convergence. For these reasons, Bayesian approaches are unsuitable for the targeted sequence capture data generated here. Although we view targeted sequence capture as more effective in terms of the degree of resolution they provide across varying depths of the actiniarian tree, we acknowledge that PCR-directed approaches remain important for understanding actiniarian diversity. At the broadest level, for superfamilial identification, the five-gene approach and targeted capture approach generally agree (see below). The notable differences are in the resolution of members of superfamily Actinostolioidea; the monophyly of this group is not clear and its diversity is poorly represented in both data sets, which complicates its interpretation using any method. At present, species-level diversity is greater within the five-gene data set, which offers advantages for some kinds of questions and suggests some benefits in terms of the analysis (reviewed by [95]). Furthermore, and importantly, the cost and technical requirements for data generation and analysis are much lower for PCR-directed approaches. The broadly similar results for questions of superfamilial identity and within-family diversity suggest that PCR-directed approaches will continue to be effective for questions at these levels.

One of the key differences between the results of this study and those of Rodríguez et al. [35] lies in the recovery of a monophyletic Acuticulata (Fig 2A and 2B). In previous studies, *Triactis* Klunzinger, 1877 [105] and *Cylista elegans* (Dalyell, 1848) [106] (as *Sagartia elegans*) were placed as part of a clade sister to the Cuticulata-Acuticulata, resulting in a non-monophyletic Acuticulata. However, consistent with genome-scale phylogenies [55,96], this tree confidently recovers Acuticulata as monophyletic, with *Triactis* resolving within Aliciidae (Fig 1; indigo). We find the same result in our five-gene tree, suggesting that the placement of *Triactis* outside Acuticulata was an artifact of the sampling employed in Rodríguez et al. [35] that is resolved when more species are included.

Additionally, a curious outlier was observed in the specimen USNM 1429254, which was collected by hand in Mo’orea, north of the old hotel on the east shore of Cook’s Bay in 2009 at a depth of 0.5 m. In the trees of Benedict et al. [55], this specimen grouped with members of the family Boloceroididae, whereas our analysis placed this sample within Aliciidae (Fig 1; indigo). These two analyses used the same bait set but included different taxa, with Benedict et al. [55] including numerous taxa for which only transcriptomic data was available. Because of the inclusion of a large proportion of transcriptomic samples in their dataset, fewer loci were recovered across the tree than in this analysis. Differences in loci and in taxonomic representation often result in varying phylogenetic placements. This sample was not included in 5-gene analyses as the sequence data were generated from target-sequence capture methods, the baits of which are not designed to retrieve the genes used in the 5-gene approach.

The phylogeny from this study also provides improved confidence in the placement of *Cylista troglodytes* and *Cylista elegans*, two species that were less confidently resolved in previous analyses (Fig 2B and 2D). Additionally, the results of this study find *Verrillactis* England, 1971 [107] closely associated with *Neoaiptasia* Parulekar, 1969 [108] and *Paraiptasia* England, 1992 [38], rather than with members of Aiptasiidae. The five-gene tree is similar to the topology of Rodríguez et al. [35] in affiliating *Verrillactis* with Aiptasiidae rather than with *Neoaiptasia* and *Paraiptasia* (Fig 2B and 2D). The clustering of *Verrillactis*, *Neoaiptasia*, and *Paraiptasia* in the target capture tree is well supported (Fig 1; mint green) and is interesting in that these three genera contain species that live on the shells of gastropod molluscs and are reported only from shallow waters of the Indo-West Pacific. In the original description of *Neoaiptasia*, Parulekar noted that it shared similarities with Aiptasiidae and with Sagartiidae, and both he and subsequent authors had to modify Aiptasiidae to align it with the diagnostic attributes of that family (see [38,108,109]). Rodríguez et al. [85] and Grajales and Rodríguez [31] were equivocal about *Neoaiptasia* and *Paraiptasia* as members of Aiptasiidae; their hesitancy was warranted, given our results.

Although both the genome-scale and five-gene trees (Fig 2C and 2D) concur in finding Sagartiidae to be non-monophyletic, the specifics of the inferred relationships are quite different. Sagartiidae has long been viewed likely to be heterogenous (e.g., [32,44,110,111]) and previous studies have uniformly failed to recover a monophyletic Sagartiidae [33,35,83,112,113] (note that Antipodactinidae, Isanthidae, Kadosactinidae, and Ostiactinidae, inferred to belong to Acuticulata in Durán-Fuentes et al. [83] are not present in this clade in other analyses). The resolution in the genome-scale analysis (Fig 1) differs from that of previous studies and from what we see in the phylogenetic reconstruction using the five-gene dataset (Fig 2D) in that Sagartiidae is reconstructed as a grade of three clades (with one having two sub-lineages). The designation of these four clades, Sagartiidae I–IV, represents a significant step forward in understanding the evolutionary relationships within this group. This finding allows for the examination of internal morphology to resurrect or describe putative families within the clade. The potential of a new system to surface similarities among taxa is the rationale for our recognition of four rather than three clades of sagartiids. We differentiate between these two subclades because we see ecological, biological, and geographic differences between Sagartiidae III and Sagartiidae IV, with Sagartiidae IV containing epibiotic species (on gastropods) of diverse genera and Sagartiidae III containing species belonging to *Cylista*. In future studies, it is likely that further morphological characters, along with molecular data, will help distinguish members of these newly identified clades and clarify the taxonomic status of Sagartiidae.

The inclusion of unidentified specimens from the Falkland Islands and their supported placement within distinct clades emphasizes the importance of sequencing known sea anemone species to build a robust genetic match database. Because anemones are notoriously difficult to identify morphologically and may contain high variability in color, pattern, and phenotypic plasticity even within a species, methods such as target capture provide an additional tool for determining diversity within a hotspot such as the South Atlantic Ocean. For the Falkland Islands specimens, the molecular identification approach demonstrated the utility of targeted sequence capture in resolving the placement of samples with ambiguous external morphology by assigning them to distinct clades with strong phylogenetic support. The overrepresentation of *Anthothoe chilensis* in this analysis reflects the inclusion of many previously unidentified specimens from the Falkland Islands that were successfully identified using this approach. These conclusions support the findings of Benedict et al. [55] for taxa from Mo’orea, where this approach proved more efficient for identification due to the sampling gaps in the BLAST database for Actiniaria and the imprecision of matches, given low sequence variability for some markers (see [114–116]). The inclusion of unidentified Falkland Islands material in this study underscores the necessity of region-specific sampling to complement global efforts. Of particular note, *Anthothoe chilensis* resolved as separate from *Anthothoe albocinta* but with *Sagartiogeton awii*, indicating the need for greater sampling and exploration into taxonomic relationships within these genera.

### Taxonomic remarks

The goals of this study included identifying families and genera requiring revision, but the level of sampling does not permit resolution of all of these issues yet. Our findings affirm monophyly of Acricoactinidae, Aliciidae, Boloceroididae, Diadumenidae, Gonactiniidae, and Metridiidae. We concur with previous studies in finding the clade containing most members of Aiptasiidae to exclude *Paraiptasia* (see [31]). Given its phylogenetic placement and the history of equivocation on its taxonomic placement, we move *Paraiptasia* to Sagartiidae, specifically noting that it is a member of Sagartiidae IV.

In addition to lacking these previously affiliated genera, we find that the least inclusive clade containing other members of Aiptasiidae also includes *Aiptasiomorpha texaensis* Carlgren & Hedgepeth, 1952 [117]. *Aiptasiomorpha texaensis* is the sole representative of Aiptasiomorphidae Carlgren, 1949 [60] included in this study. While the finding of a close relationship between *A. texaensis* and *E. diaphana* (see Fig 1; cerulean) suggests a close phylogenetic relationship between these taxa, further investigation is required to understand how to represent this taxonomically. The genus *Aiptasiomorpha* Stephenson, 1920 [32] is not well characterized: *Aiptasiomorpha minima* (Stephenson, 1918) [118], type species of the genus, has previously been placed in *Aiptasia* and *Diadumene* (see [118]), and the definition of *Aiptasiomorpha* could potentially apply to many members of acuticulata, raising questions about its monophyly and priority. Genera within Aiptasiidae are not clearly differentiated, being either very broad (e.g., *Aiptasia*) or very narrow (e.g., *Bartholomea*, *Laviactis*) (see [31,119]). Further study of this group is needed to determine whether *Aiptasiomorpha* (and thus Aiptasiomorphidae) is a junior synonym of Aiptasiidae or whether *Aiptasiomorpha* is heterogenous, with its constituent species having affiliations to multiple acuticulate groups.

The inclusion of key type material, such as *Aiptasimorpha minima, Neoaiptasia commensali* Parulekar, 1969 [108] and *Sagartia viduata* (Müller, 1776) [44,120], will be essential for making the necessary taxonomic revisions that can stabilize the nomenclature within the acuticulate clade. Without these critical specimens, it remains challenging to confidently synonymize taxa or assign names to the appropriate family-level groups of Sagartiidae I–IV. Because we lack clarity on the position of these key taxa, we do not designate any clade here as true Sagartiidae or propose new names for the four clades we recognize within Sagartiidae. The family name will likely remain with clade containing the type species of Sagartiidae, *Sagartia viduata*.

The placement of the following three families remains unresolved: Haliactinidae, Haliplanellidae, and Sagartiomorphidae. The absence of members of these families in this analysis leaves room for the topology to adjust in future analyses, as more comprehensive taxon sampling is conducted. Further phylogenetic analyses, incorporating type material and members of the families Haliactinidae, Haliplanellidae, and Sagartiomorphidae, will be necessary to resolve the remaining taxonomic uncertainties. These unsampled lineages are imperative for a thorough phylogeny of Acuticulata and to support a much-needed revision of Sagartiidae. Together, this will help stabilize the taxonomy of Acuticulata, which will provide a clearer framework for future research into this diverse and ecologically significant clade.

## Supporting information

S1 Appendix Metadata for samples included in target capture phylogenetic analysis. This appendix provides metadata for all samples used in the target sequence capture analysis. Information for each sample includes: family (as recovered in this analysis), genus, species (if applicable), sample code, voucher location, sequences provided by (if a sample was not sequenced in the sequencing run conducted by the authors of this study), sample provided by, locality information, notes. Samples listed as they appear (top to bottom) in Fig 1.
(XLSX)

S1 Fig Phylogenetic reconstruction of Acuticulata using a five-gene dataset. Maximum likelihood phylogenetic tree based on concatenated sequences from two nuclear (18S, 28S) and three mitochondrial (12S, 16S, *cox*3) genes for 99 samples. Ultrafast bootstrap values as shown at nodes. Families resolved as monophyletic color-coded by family-level grouping as labeled in the figure. Samples in red text represent outliers that did not align with family-level groupings as expected prior to the findings of this study.
(TIF)

S2 Fig Neighbor-joining phylogenetic reconstruction of Acuticulata using 50% occupancy matrix of target capture loci. Taxa color-coded by family-level grouping: Metridiidae – red, Acricoactinidae – orange, Diadumenidae – yellow, Sagartiidae I – lime green, Sagartiidae II – kelly green, Sagartiidae III – forest green, Sagartiidae IV – mint green, Aiptasiidae – cerulean, Aliciidae – indigo, Gonactiniidae – violet, Boloceroididae – fuchsia.
(TIF)
