# Supplementary figures and images for: Resolving Acuticulata (Metridioidea: Enthemonae: Actiniaria), a clade containing many invasive species of sea anemones
Source: PLoS One. 2025 Aug 14;20(8):e0328544. doi: 10.1371/journal.pone.0328544 (PMC12352762; doi:10.1371/journal.pone.0328544)

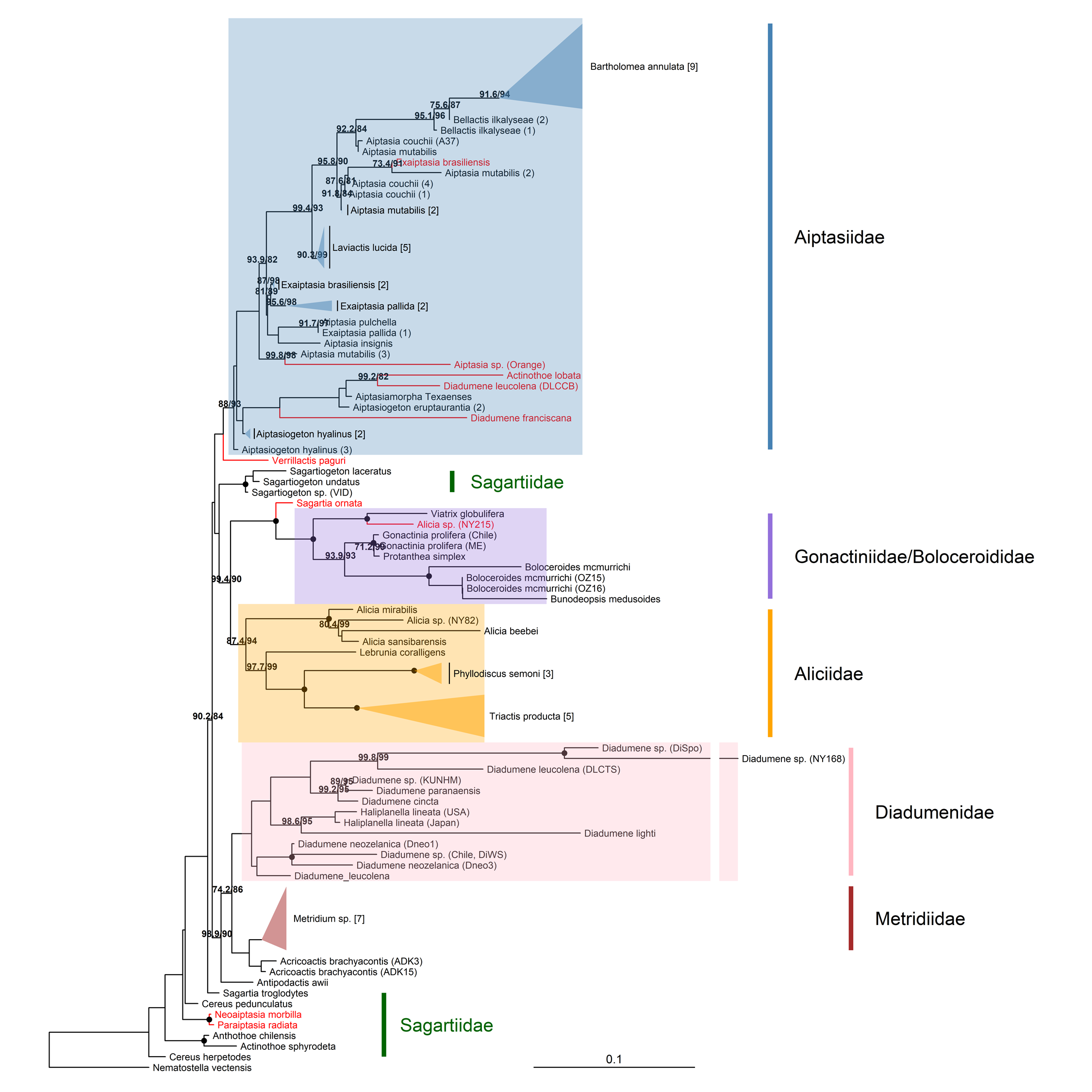

Supplement: S1 Fig — Maximum likelihood phylogenetic tree based on concatenated sequences from two nuclear (18S, 28S) and three mitochondrial (12S, 16S, cox3) genes for 99 samples. Ultrafast bootstrap values as shown at nodes. Families resolved as monophyletic color-coded by family-level grouping as labeled in the figure. Samples in red text represent outliers that did not align with family-level groupings as expected prior to the findings of this study. (TIF) [file pone.0328544.s002.tif]

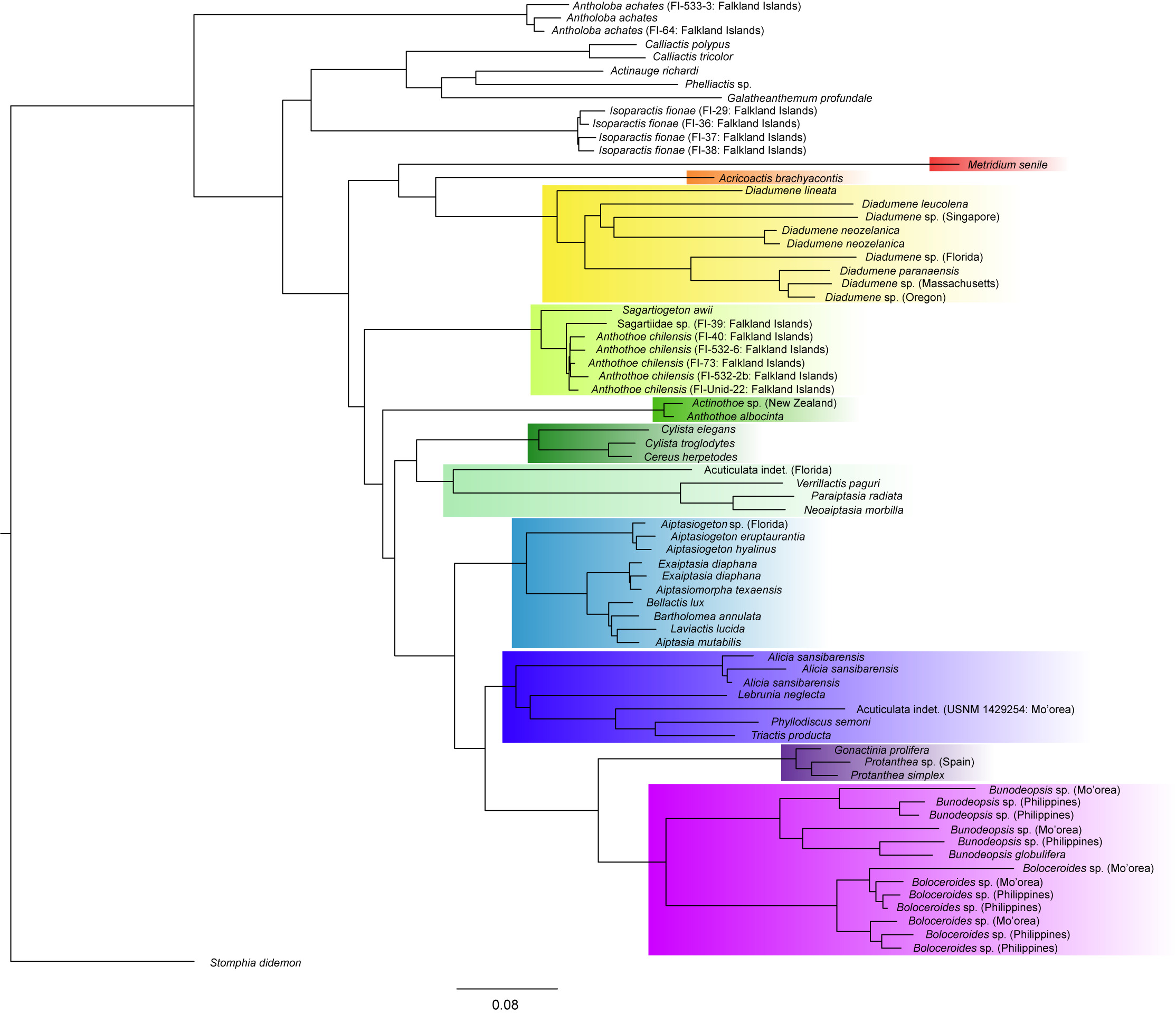

Supplement: S2 Fig — Taxa color-coded by family-level grouping: Metridiidae – red, Acricoactinidae – orange, Diadumenidae – yellow, Sagartiidae I – lime green, Sagartiidae II – kelly green, Sagartiidae III – forest green, Sagartiidae IV – mint green, Aiptasiidae – cerulean, Aliciidae – indigo, Gonactiniidae – violet, Boloceroididae – fuchsia. (TIF) [file pone.0328544.s003.tif]
